# Roles of the Default Mode and Multiple-Demand Networks in Naturalistic versus Symbolic Decisions

**DOI:** 10.1523/JNEUROSCI.1888-20.2020

**Published:** 2021-03-10

**Authors:** Verity Smith, John Duncan, Daniel J. Mitchell

**Affiliations:** ^1^Medical Research Council Cognition and Brain Sciences Unit, University of Cambridge, Cambridge CB2 7EF, United Kingdom; ^2^Department of Experimental Psychology, University of Oxford, Oxford OX2 6GG, United Kingdom

**Keywords:** cognitive control, context, default mode network, fMRI, multiple demand

## Abstract

The default mode network (DMN) is often associated with representing semantic, social, and situational content of contexts and episodes. The DMN may therefore be important for contextual decision-making, through representing situational constraints and simulating common courses of events. Most decision-making paradigms, however, use symbolic stimuli and instead implicate cognitive control regions, such as the multiple demand (MD) system. This fMRI study aimed to contrast the brain mechanisms underlying decision-making based on rich naturalistic contexts or symbolic cues. While performing an ongoing task, 40 human participants (25 female) responded to different sounds. For one sound, the stimulus-response mapping was fixed; responses for the other sounds depended on the visual context: either lifelike scenes or letter symbols, varying across participants. Despite minimal behavioral differences between the groups, posterior DMN regions showed increased activity during context-dependent decision-making using the naturalistic scenes only, compared with symbolic cues. More anterior temporal and frontal DMN regions showed a different pattern, with sensitivity to the need for contextual control, but not to the type of context. Furthermore, in the scenes group, widespread DMN regions showed stronger representation of not just the context but also the sound whose significance it modulated. In comparison, the MD system showed strong univariate activity for every decision, but, intriguingly, somewhat reduced activity in the case of a scene-based but demanding context-dependent decision. Depending on context, we suggest, either DMN or MD regions may play a prominent role in selection and control of appropriate behavior.

**SIGNIFICANCE STATEMENT** Contextual knowledge is widely believed to be important for guiding real-world goal-directed behavior. Much remains to be understood, however, regarding the underlying brain mechanisms. Using a novel paradigm to contrast decisions based on richly meaningful naturalistic scenes with decisions based on symbolic cues, we find that both multiple demand regions and default mode regions may contribute to the cognitive control of behavior. Rich semantic context enhances representation not just of the context itself, but also of the contents of the decision that it controls. Dependence of a decision on naturalistic context can also reverse the common pattern of multiple demand regions responding more, and default mode regions responding less, to more difficult decisions.

## Introduction

Spatial, semantic, and social context shapes everyday cognition. Context can help us understand ambiguous sentences, organize information for memory encoding, or understand a person's actions. The behavioral benefit of contextual information has been studied for decades, particularly in memory recall and language comprehension (Bransford and Johnson, 1972; Godden and Baddeley 1975; Zwaan and Radvansky, 1998; Spivey et al., 2002).

Context is also important for effective goal-directed behavior. Theories propose contextual schemas, acquired through repeated experiences in similar contexts, can guide future behaviors by representing situational constraints and simulating common courses of events (Bar, 2007, 2009; Zacks et al., 2007; Ranganath and Ritchey, 2012). Levels of contextual representation can include generalized, semantic information (you must be quiet in libraries), to more specific knowledge of a particular place (in this library study room, you can whisper) or episodic knowledge of a specific event (at this open day, you can talk).

Much remains to be understood, however, regarding the underlying brain mechanisms. Because of its association with scene and context representation (Hassabis and Maguire, 2007; Baldassano et al., 2016, 2017; Chen et al., 2017), and memory retrieval (Vilberg and Rugg, 2012; Richter et al., 2016), researchers have suggested the default mode network (DMN) may be critical for context-guided cognition (Bar, 2007, 2009; Ranganath and Ritchey, 2012). Ranganath and Ritchey (2012) suggested that DMN regions, particularly the parahippocampus (PHC), retrosplenial cortex (Rsp), posterior cingulate cortex (PCC), and angular gyrus, form a “posterior medial system,” which represents a situation model based on the current situational context, including its social, semantic, and temporal associative relationships. Despite deactivation during many “executive” tasks, DMN regions are consistently active during studies using naturalistic stimuli, such as movies, scenes, and personal events (Addis et al., 2007; Andrews-Hanna et al., 2010; Baldassano et al., 2017) and, recently, have been implicated in integration of spatial and emotional information (Lanzoni et al., 2020) and memory-guided cognition (Vatansever et al., 2017; Murphy et al., 2018). Rich, meaningful stimuli, often used when studying the DMN, allow access to semantic and social associations developed over time from other experiences of similar environments. For example, after entering a restaurant, we might predict that someone would then ask for a table. Plausibly, the DMN is important for context-dependent decision-making, but primarily when the context is associatively rich, allowing use of episodic and semantic knowledge to make predictions about upcoming events (Bar, 2007, 2009; Zacks et al., 2007; Ranganath and Ritchey, 2012).

Despite these proposals, there is little direct evidence implicating the DMN in context-dependent cognitive control. Cognitive control is more typically associated with the multiple-demand (MD) system. MD activity is associated with many types of cognitive demand, including working memory load, response competition, and complexity of stimulus-response rules (Duncan and Owen, 2000; Fedorenko et al., 2013). Often, DMN and MD networks show complementary patterns of activity: whereas MD activity increases during many tasks compared with rest, DMN activity decreases (McKiernan et al., 2003, 2006; Fox et al., 2005). On these grounds, we might expect MD, rather than DMN, regions to be involved in active decision-making.

Here we address the roles of DMN and MD networks in different forms of “contextual” control. We directly compared activity during contextual decision-making tasks using symbolic cues and richly meaningful scenes. In this way, we aimed to test whether the DMN is specifically important during meaningful context-dependent decision-making. In line with previous theories, DMN regions were predicted to be active during context-dependent decision-making with meaningful contexts compared with when using symbolic cues. As MD activity is associated with diverse task demands (Duncan and Owen, 2000; Fedorenko et al., 2013), the MD network was predicted to be active for context-dependent decision-making regardless of the semantic richness. Then, using multivariate representational similarity analysis (RSA), we examined task-related content represented within these networks during scene and letter versions of the task, again predicting that the DMN would show a relative enhancement of representation for scene versus letter contexts.

## Materials and Methods

### 

#### 

##### Participants

Forty-eight participants (29 female), between 18 and 35 years of age, were recruited through the Medical Research Council Cognition and Brain Sciences Unit participant panel. All participants selected were right-handed, native English speakers, with normal or corrected-to-normal vision. The study was conducted in accordance with ethical approval from the Cambridge Psychology Research Ethics Committee. Eight participants (4 female) were excluded from further analysis: 4 because of poor task performance (≤50% correct sound responses in one or more task condition), 3 because of excessive motion (over 5 mm translation), and 1 who did not complete the experiment.

As the task used a between-subjects contrast, the participants were split into two groups of 20 participants: scenes (14 females, mean age: 24.1 years, SD: 3.7 years) and letters (12 females, mean age: 23.7 years, SD: 4.8 years). The ages of the two groups did not significantly differ (*t*_(38)_ = 0.31, *p* = 0.76, *d* = 0.10, 95% CI = −0.54, 0.74]).

##### Task

**Figure 1.**
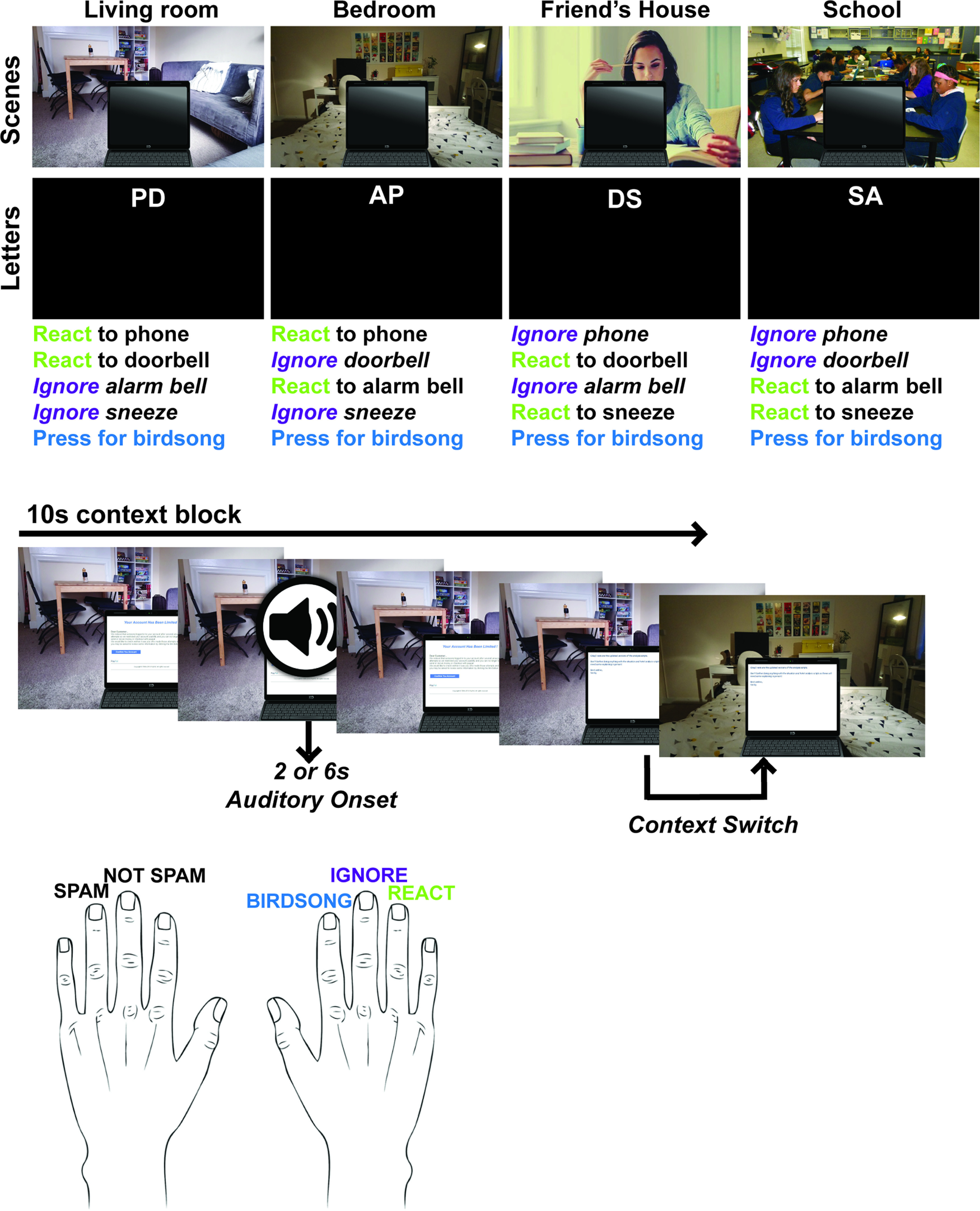
Task design. The ongoing task required participants to read emails and judge whether they were spam or not by left hand button press. Background scenes or letters were presented in 10 s blocks. On 75% of blocks, the background context was different from the previous context; on the other 25% of the blocks, the background context was repeated. An auditory stimulus was played at either 2 or 6 seconds into each block. Participants were asked to respond to the sound by right hand button press. For birdsong, the response button was fixed as the right index finger. Correct responses for the other four sounds were context-dependent. For the scenes group, each of the four visual scenes indicated a situational context, with details of the place, what they would be doing, and the time of day. Using the situational information, participants were asked to indicate whether they would “ignore” (indicated by middle finger response) or “react to” (ring finger) the sounds, based on the rules presented above. For the letters group, the experimenter showed participants that the first letter of each context-dependent sound corresponded to the possible letters in the on-screen letter pairs. If the sound matched one of the letters in the letter pair, then participants were asked to press “react”; otherwise, they were asked to press “ignore.”

Task events are illustrated in Figure 1. To give participants an immersive, ongoing task to focus on, they were presented with emails and asked to categorize each email as either spam or not spam by left hand ring and middle finger button presses, respectively. This task was nonspeeded, and each email remained on the screen until participants made a response. Equal numbers of spam and nonspam emails were presented to participants in a randomized order. The emails were presented on one of four backgrounds in blocks of 10 s. 20 participants received scene backgrounds (the “scenes” group), and the other 20 participants saw black backgrounds with two white letters displayed centrally at the top of the screen (the “letters” group). The two letters were always presented in the same order for each of the four conditions (e.g., always PD, never DP). The eight different backgrounds are presented in Figure 1.

At either at 2 or 6 s after the start of each 10 s block, a sound played and participants were asked to respond to it as quickly as possible. Participants were told to prioritize the auditory task, so that usually, when a sound occurred, they would interrupt processing of the email, respond to the sound, then return to the email. However, sound and email responses were allowed in either order, with each email remaining until the response was given, and each sound remaining until either response or the end of the block. Both the scene and letters groups heard the same four context-dependent sounds (doorbell, phone, sneeze, and alarm bell), which required participants to use the background information (scenes or letters) to decide which button to press, and one context-independent sound (birdsong), with the same response regardless of context.

For the scenes group, each visual scene cued one of four situational contexts: alone in your living room in the middle of the day, alone in your bedroom having just woken up, studying at a friend's house in the evening while waiting for pizza delivery, and working at school waiting for the end of the lesson. Participants were asked to use this situational information to decide whether they would “react to” or “ignore” context-dependent sounds. “React” was indicated by pressing a key with the ring finger of the right hand, while “ignore” was indicated with the right middle finger. The rules for each context are presented in Figure 1. In the living room, participants were told to react to the phone and doorbell. They were told to ignore the sneeze (no need to say anything as they were alone) and the alarm bell (alarm clock irrelevant in the middle of the day; in this case, “ignoring” was taken to mean simply switching the alarm off). Similar explanations were given for the other three contexts. In the bedroom, participants were to react to the phone and alarm bell (signaling time to get up), but to ignore sneeze and doorbell. When studying at a friend's house, participants were told to ignore phone call or alarm bell, but react to sneeze (e.g., apologize) and doorbell (answer the door for pizza). Finally, at school, the participants were asked to ignore doorbell or phone, but to react to sneeze (apology) or alarm bell (indicating the end of the lesson).

For the letters group, participants were shown that each letter at the top of the screen corresponded to the first letter of one of the sounds (P, phone; D, doorbell; A, alarm bell; S, sneeze). When a sound played, if the beginning letter of the sound was present in the background letter pair, then participants were asked to press to “react” to the sound. If the beginning letter of the sound was not present, then participants were asked to press “ignore.” As shown in Figure 1, the background letters were paired such that the four pairs matched the rules for the scenes group (e.g., letter context “PD” = scene context “living room”).

The 10 s blocks were presented in a pseudorandom order. On 75% of blocks, the background context changed; and on 25% of the blocks, it repeated. These background repeats as well as the jittered sound onset time were implemented to help separate fMRI responses to sound onset and background switch. The experiment consisted of three runs of 97 blocks. As transient DMN activity can accompany large cognitive transitions (Crittenden et al., 2015; Smith et al., 2018), the first block of each run (background context and sound randomized, sound onset at 6 s) was discarded from analysis. Following this first block, each run contained 24 blocks of each of the background contexts. Within each background, each context-dependent sound was played 4 times per run, and birdsong was played 8 times per run, with half of the sounds in each case presented at 2 s and half at 6 s after block onset. Each run also contained 30 s rest periods at the start and end.

The 300 spam and 300 nonspam email stimuli, stripped of personally identifiable details, were sourced from the first author's emails. To ensure participants were reading the emails rather than remembering their previous response to the same email, in each run a new set of emails (100 spam and 100 nonspam) were used. Email stimuli measured ∼7.5 (width) × 5.6 (height) degrees of visual angle, and were positioned ∼1.8 degrees of visual angle below the center of the screen. In the scenes group, this positioning fit the emails inside a superimposed laptop to convey the impression that the participants were situated within the scene while checking their emails; the scene image spanned the remainder of the screen (visual angle ∼25 × 14 degrees). In the letters group, the letter cues were positioned 4.6 degrees of angle above the center of the screen. The letter stimuli measured ∼2.3 (width) × 1.5 (height) degrees of visual angle. The experiment was controlled using Psychophysics Toolbox (Brainard, 1997) for MATLAB (The MathWorks).

##### Training

Participants were carefully pretrained to ensure good learning of the task rules. First, participants were introduced to the email task and learned the spam and nonspam button presses. Then participants were introduced to the sounds. First, they heard the birdsong and learned the corresponding button press. Then participants were asked to identify the four context-dependent sounds by name, and corrected if they had any difficulty. Participants were then introduced to each context and the associated task rules. For the scenes group, the experimenter elaborated on each of the contexts and explained why the response rules applied. For the letters group, the experimenter explained the match between the letter contexts and names of the context-dependent sounds. Participants were then presented with each sound in every context in a pseudorandom order and asked to say what button they would press. When the experimenter was sure that the participant had understood and memorized the task rules, participants moved on to complete a 12-block practice version of the task outside of the scanner.

To ensure that participants could read the emails in the scanner, once in the scanner, but before running the tasks, participants were presented with an example email and asked to read it out loud.

##### fMRI data acquisition

Images were acquired using a 3 T Siemens Prisma MRI scanner, fitted with a 32-channel head coil. fMRI acquisitions used T2*-weighted multiband EPI (multiband acquisition factor 3 for 2.5 mm slices with no interslice gap, TR 1.1 s, TE 30 ms, flip angle 62°, voxel size 2 × 2 mm^2^). T1-weighted multiecho MPRAGE images were also obtained (TR 2.53 s, TEs 1.64, 3.5, 5.36 and 7.22 ms, flip angle 9°, voxel size 1 mm^3^).

##### fMRI preprocessing

Images were preprocessed using automatic analysis (version 4) (Cusack et al., 2015) and SPM 12 (Wellcome Department of Cognitive Neurology, London) for MATLAB (The MathWorks). Preprocessing involved spatial realignment of the raw EPIs, slice-time correction to the middle slice, coregistration of the functional EPI images to the structural T1-weighted image, and normalization to the MNI template brain. For the whole-brain, voxelwise, univariate analysis only, functional images were spatially smoothed using a Gaussian kernel of 10 mm FWHM.

##### ROIs

To construct DMN regions, we began from the 20 coordinates defined by Andrews-Hanna et al. (2010), grouped by them into core, medial temporal lobe (MTL), and dorsomedial PFC (dmPFC) subnetworks. In Andrews-Hanna et al. (2010), DMN ROIs were defined as 8 mm spheres around the central coordinates presented in Table 1. Here these ROIs were expanded so as to encompass a brain volume more representative of the typical full DMN (Wen et al., 2020).

**Table 1.** Central MNI coordinates for DMN regions defined by Andrews-Hanna et al. (2010)^a^

Core DMN	*x*	*y*	*z*	MTL DMN	*x*	*y*	*z*	dmPFC DMN	*x*	*y*	*z*
L PCC	−8	−56	26	L pIPL	−44	−74	32	L TPJ	−54	−54	28
R PCC	8	−56	26	R pIPL	44	−74	32	R TPJ	54	−54	28
L amPFC	−6	52	−2	L Rsp	−14	−52	8	L LTC	−60	−24	−18
R amPFC	6	52	−2	R Rsp	14	−52	8	R LTC	60	−24	−18
				L PHC	−28	−40	−12	L TempP	−50	14	−40
				R PHC	28	−40	−12	R TempP	50	14	−40
				L HF	−22	−20	−26	dmPFC	0	52	26
				R HF	22	−20	−26				
				vmPFC	0	26	−18				

^a^Abbreviations as in Table 2.

For this purpose, DMN masks were generated using networks 10, 15, 16, and 17 from the 17 network cortical parcellation reported in Yeo et al. (2011). Networks 15, 16, and 17 closely corresponded to the three DMN networks described by Andrews-Hanna et al. (2010). Network 10 was described by Yeo et al. (2011) as the orbital frontal-temporopolar network but was included as it contained the ventromedial PFC (vmPFC) coordinate from Andrews-Hanna et al. (2010). These four networks were combined and then smoothed with a 4 mm FWHM Gaussian kernel, and voxels with values > 0.5 after smoothing were retained. The combined network was then parcellated into 20 subregions by assigning each voxel to its closest DMN coordinate as defined by Andrews-Hanna et al. (2010). In cases where noncontiguous clusters were assigned to the same coordinate, only the closest cluster with size > 45 voxels was retained. To make all ROIs bilateral, left and right volumes for each region (e.g., left and right PCC) were concatenated to generate 11 bilateral ROIs: PCC, anterior medial PFC (amPFC), PHC, hippocampus (HF), Rsp, posterior inferior parietal lobe (pIPL), vmPFC, lateral temporal cortex (LTC), temporoparietal junction (TPJ), temporal pole (TempP) and dmPFC. The resulting full set of DMN ROIs is shown in Figures 4 and 7.

**Table 2.** Results of two-way mixed-model ANOVAs with a between-participant factor of group and a within-participant factor of context dependence, for each ROI^a^

Network	ROI	Context dependence			Group			Interaction		
		*F*_(1,38)_	*p*	η_p_^2^	*F*_(1,38)_	*p*	η_p_^2^	*F*_(1,38)_	*p*	η_p_^2^
Core DMN	PCC	1.39	0.25	0.04	4.66	0.04	0.11	5.13	0.03	0.12
	amPFC	7.62	0.01	0.17	0.02	0.90	4.1e-4	1.91	0.18	0.05
MTL DMN	pIPL	1.10	0.30	0.03	7.19	0.01	0.16	14.33*	5.3e-4	0.27
	Rsp	1.73	0.12	0.04	16.96*	2.0e-4	0.31	25.96*	1.0e-5	0.41
	PHC	0.91	0.35	0.02	16.92*	2.0e-4	0.31	40.64*	1.7e-7	0.52
	HF	4.98	0.03	0.12	7.94	7.6e-3	0.17	10.44*	2.6e-3	0.22
	vmPFC	12.16*	1.3e-3	0.24	1.24	0.27	0.03	1.82	0.19	0.05
dmPFC DMN	TPJ	3.99	0.05	0.10	2.24	0.14	0.06	0.88	0.35	0.02
	LTC	15.30*	3.7e-4	0.29	0.22	0.64	5.8e-3	0.01	0.93	2.2e-4
	TempP	7.61	8.9e-3	0.17	1.68	0.20	0.04	0.71	0.41	0.02
	dmPFC	7.27	0.01	0.16	0.09	0.77	2.3e-3	0.02	0.89	5.4e-4
MD	IPS	3.08	0.09	0.07	0.68	0.41	0.02	11.41*	1.7e-3	0.23
	pdLFC	0.96	0.33	0.02	0.19	0.66	5.1e-3	5.56	0.02	0.13
	IFJ	2.99	0.09	1.6e-3	0.02	0.99	1.0e-3	1.13	0.29	0.13
	AI/FO	0.51	0.48	0.01	0.33	0.57	8.5e-3	2.07	0.16	0.05
	pIFS	0.82	0.37	0.02	0.21	0.65	5.6e-3	0.47	0.50	0.01
	aIFS	0.06	0.80	0.07	0.04	0.85	4.0e-6	5.75	0.02	0.03
	preSMA/ACC	0.13	0.72	3.4e-3	0.51	0.48	0.01	3.64	0.06	0.09

^a^CoreDMN:PCC, posterior cingulate cortex; amPFC, anteromedial prefrontal cortex; MTLsubnetwork: pIPL, posterior inferior parietal lobe; Rsp, retrosplenial cortex; PHC, parahippocampal cortex; HF, hippocampal formation; vmPFC, ventromedial prefrontal cortex; dMPFC subnetwork: TPJ, temporo-parietal junction; LTC, lateral temporal cortex; TempP, temporal pole; dmPFC, dorsomedial prefrontal cortex; MD: IPS, inferior parietal sulcus; pdLFC, posterior dorsolateral frontal cortex; IFJ, inferior frontal junction; AI/FO, anterior insula/frontal operculum area; pIFS, posterior inferior frontal sulcus; aIFS, anterior inferior frontal sulcus; preSMA/ACC, pre-supplementary motor/anterior cingulate cortex.

*Significant effects after Holm-Bonferroni multiple correction comparison across ROIs within each network (whole DMN, MD).

Frontoparietal MD ROIs were based on data from Fedorenko et al. (2013), divided into subregions as described in Mitchell et al. (2016). MD regions (see Figs. 5, 8) included the posterior–anterior extent of the inferior frontal sulcus, a posterior dorsal region of lateral PFC, inferior frontal junction (IFJ), anterior insula/frontal operculum, presupplementary motor area/dorsal anterior cingulate, and intraparietal sulcus (IPS). A template for these regions was downloaded from http://imaging.mrc-cbu.cam.ac.uk/imaging/MDsystem. Only the frontoparietal ROIs were selected.

Finally, as the MD volume anterior insula/frontal operculum and the DMN volume vmPFC showed slight overlap, the region of overlap was removed from both ROIs.

##### Univariate analysis

Data for each participant were examined using the GLM, conducted in SPM 12 (Wellcome Department of Cognitive Neurology, London) for MATLAB (The MathWorks). Our model included regressors for the auditory task, along with separate regressors for the onset of each 10 s block. Activity from the rest of the block, corresponding to time spent doing the ongoing email task, was left as the implicit baseline. Regressors for the auditory task were separately created for each combination of sound (doorbell, phone, alarm bell, sneeze, birdsong) by context (living room, bedroom, friend's school, or PD, AP, DS, SA). Each regressor was modeled as an event from sound onset to offset (either response, or the end of the block), convolved with the canonical HRF. Regressors for block onsets were modeled for each combination of context switch type (context switch, context stay) by context (living room, bedroom, friend's school, or PD, AP, DS, SA). These block onset regressors were modeled as δ functions convolved with the canonical HRF. The 30 s rest periods at start and end of each run were modeled separately and then discarded from the main analysis.

For each participant, mean β values for each combination of sound and context, compared with implicit baseline, were extracted from each ROI using the MarsBaR toolbox (Brett et al., 2002). These were averaged to give a single mean contrast of context-dependent sounds (doorbell, phone, alarm bell, and sneeze) > implicit baseline. A contrast was also constructed for context-independent birdsong > implicit baseline. To examine the effects of context dependence between groups, a mixed-model ANOVA was constructed with context dependence as the within-participant factor (context-dependent > implicit baseline and context-independent > implicit baseline) and group as the between-participant factor (scenes and letters). For the first analysis, an additional within-participant factor of ROI was included for each network separately. Further ANOVAs examined the effect of context dependence between groups for each ROI separately. A similar ANOVA was used in a whole-brain voxelwise analysis, thresholded at *p* < 0.05 corrected for multiple comparisons using the false discovery rate (FDR).

##### Multivariate analysis

To understand the representational content in DMN and MD regions, an RSA was performed, using linear discriminant contrast (LDC) (Nili et al., 2014) as the measure of dissimilarity between activation patterns. The LDC (also known as the “crossnobis” estimator) is a cross-validated estimate of the squared Mahalanobis distance between two patterns (Walther et al., 2016). True distances would be positively biased as noise adds dissimilarity between patterns of activity. The crossnobis estimate is calculated using leave-one-run-out cross-validation to give a distance estimate that is distributed around zero for identical but noisy patterns, and is not positively biased. The analysis used the RSA toolbox (Nili et al., 2014), in conjunction with in-house software. This analysis was based on the same design matrix as the standard GLM described above. For each participant, unsmoothed voxelwise activity patterns were obtained for each ROI during auditory events for each combination of sound by context, resulting in 20 patterns for each run. Block onset activity for each combination of context and switch type was also modeled. The LDC dissimilarity between pairs of response patterns (e.g., doorbell-bedroom vs phone-bedroom) was calculated over pairs of runs or folds (i.e., 1–2, 2–3, 1–3). For each fold, one run was first assigned as the testing set (e.g., Run 1) and the other as the training set (e.g., Run 2). The pairs of patterns from the testing run (Run 1) were projected onto the linear discriminant fit to the training run (Run 2), and the difference between the projected patterns (distance along the discriminant) was calculated. This process was also conducted in reverse (e.g., Run 2 patterns projected onto a linear discriminant fitted from Run 1), and an average dissimilarity calculated across both directions and folds. To compare dissimilarities across ROIs of different sizes, the LDC values were normalized by dividing by the number of voxels in each ROI. This resulted in a 20 × 20 symmetrical representational dissimilarity matrix (RDM) for each ROI of each participant.

**Figure 2.**
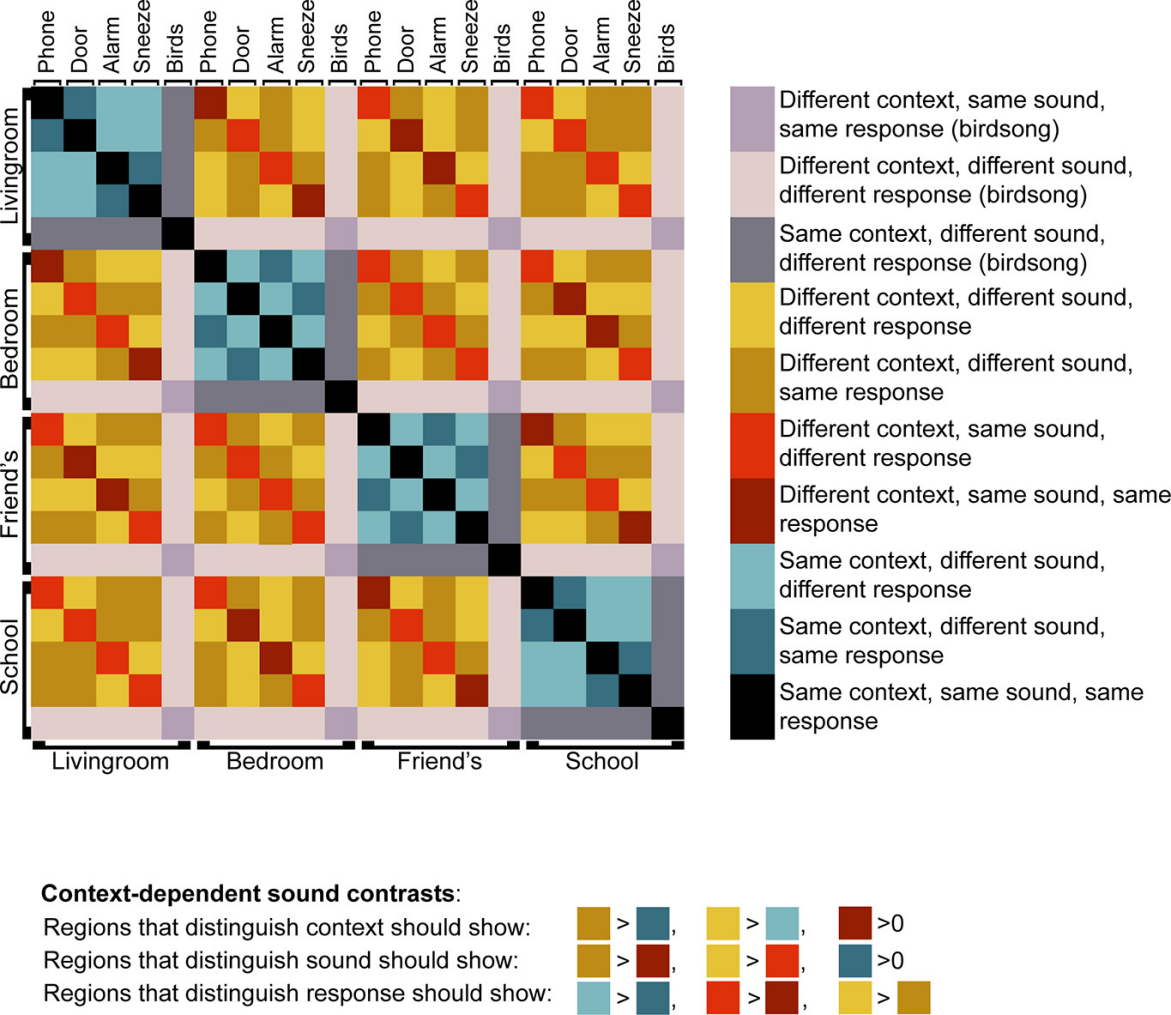
The model RDM for the scene context group. Each square represents dissimilarity between pairs of auditory events. Blue squares represent different context-dependent sounds within the same context. Red squares represent the same context-dependent sounds in different contexts. Yellow squares represent different context-dependent sounds in different contexts. For all colors, correct-response similarity (same = both react, or both ignore; different = one react, one ignore) is reflected by luminance where darker colors represent same response and lighter colors represent different responses. Gray represents dissimilarity measures between and within birdsong events. Diagonal entries (black squares) are zero by definition as they do not reflect dissimilarities between different events. Below the matrix are the contrasts used to examine context, sound, and response decoding. An equivalent matrix was constructed for the letter context group.

The model RDM for scene contexts (equivalent for letters) is shown in Figure 2. Blue squares represent different context-dependent sounds within the same context. Red squares represent the same context-dependent sounds in different contexts. Yellow squares represent different context-dependent sounds in different contexts. For all colors, correct-response similarity (same = both “react,” or both “ignore”; different = one “react,” one “ignore”) is reflected by luminance, where darker colors represent same response and lighter colors represent different responses. Gray represents dissimilarity measures within birdsong events or between birdsong and context-dependent sounds. Diagonal entries (black squares) are zero by definition as they do not reflect dissimilarities between different events. Based on this matrix, separate contrasts were used to examine coding of context, sound, and response. Figure 2 also presents the examined contrasts. Contrasts were constructed just for the context-dependent sounds, where sounds, contexts, and responses were balanced. If regions distinguish between contexts, dissimilarity should be greater for different sounds in different contexts (yellow) compared with in the same context (blue); and dissimilarity for different contexts should also be significantly greater than zero even when sound and response are the same (dark red). If regions can distinguish between sounds, dissimilarity should be greater for different sounds in different contexts (yellow) compared with the same sound in different contexts (red); and dissimilarity for different sounds should also be significantly greater than zero even when context and response are the same (dark blue). If regions can distinguish between response type, dissimilarity should be greater for different responses (light colors) compared with the same response (dark colors), when other differences are matched. Contrasts were computed by averaging dissimilarity values across each color for each ROI in each participant; and then, for each ROI and for each decoding measure (context, sound and response), the three relevant contrasts were averaged. Greater than chance decoding of each information type was tested using one-tailed *t* tests against zero, and two-tailed *t* tests were used to compare decoding between conditions.

##### RT dissimilarity

In multivariate analysis, different patterns of brain activity for two task events may in part reflect (within-participant) differences in reaction time (RT) (Todd et al., 2013; but see Woolgar et al., 2014). To examine this possibility, we repeated the RSA analyses with RT data. For each participant, mean RTs were obtained for auditory events in each combination of sound by context, resulting in 20 mean RTs per run. RT differences between pairs of auditory events (e.g., doorbell-bedroom and phone-bedroom) were then calculated per person per run, resulting in a 20 × 20 matrix of RT differences per person, per run. Mirroring the LDC measure of dissimilarity used in the RSA, RT differences were then cross-validated over pairs of runs, or folds (i.e., 1–2, 2–3, 1–3) by element-wise multiplication of the signed RT difference matrices from the two runs per fold. Finally, a mean RT dissimilarity was calculated by averaging across all the folds in both directions. This resulted in a 20 × 20 symmetrical matrix for each participant, equivalent to the RDM presented in Figure 2. Based on this matrix, the contrasts presented in Figure 2 could be performed for the within-subject RT difference data as well as the multivariate brain data.

##### Code accessibility

Code and data are available on request. ROI masks and univariate statistical maps are available from Neurovault (https://neurovault.org/collections/9224/).

## Results

### Behavior

For the email task, independent-samples *t* tests found no difference between performance in the scene and letters groups in either error (M_scene_=14.2%, M_letter_=14.9%, *t*_(38)_ = 0.31, *p* = 0.76, *d* = 0.09, 95% CI = [−0.54, 0.74]) or RT (M_scene_=1.69s, M_letter_=1.65s, *t*_(38)_ = 0.51, *p* = 0.61, *d* = 0.16, 95% CI = [−0.48, 0.80]).

**Figure 3.**
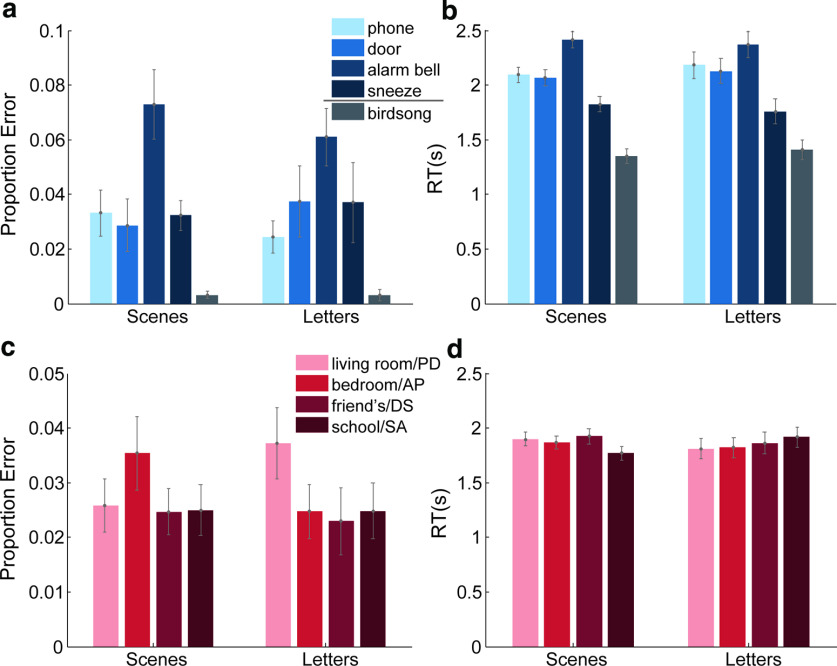
Auditory task performance. (***a***) Proportion error and (***b***) RT during auditory task performance for each sound type × group. (***c***) Proportion error and (***d***) RT during auditory task performance for each context type × group. Error bars indicate SEM across participants.

For the auditory task, Figure 3*a*, *b* presents performance broken down by groups and sound types. For both proportion error and RT, a strong effect of sound type is seen. Participants made fewer errors and were faster to respond during context-independent (birdsong) trials compared with context-dependent sound trials. Participants also made more errors and were slower to respond to the alarm bell than to the other context-dependent sounds. A two-way mixed-model ANOVA with group as the between-participant factor (scenes and letters) and sound type as the within-participant factor (phone, door, alarm bell, sneeze, and birdsong) confirmed these impressions. A significant main effect of sound type on proportion error (*F*_(4,152)_ = 12.25, *p*= 1.2e-8, η_p_^2^ = 0.24) and RT (*F*_(4,152)_ = 154.7, *p* = 1.6e-52, η_p_^2^ = 0.80) was found. Neither the effect of group (proportion error: *F*_(1,38)_ = 0.05, *p* = 0.83, η_p_^2^ = 1.2e-3; RT: *F*_(1,38)_ = 0.03, *p* = 0.87, η_p_^2^ = 7.4e-4) nor the interaction between group and sound type (proportion error: *F*_(4,152)_ = 0.46, *p* = 0.77, η_p_^2^ = 0.01; RT: *F*_(4,152)_ = 1.25, *p* = 0.29, η_p_^2^ = 0.03) was significant.

Figure 3*c*, *d* presents auditory performance broken down by groups and task contexts. A two-way mixed-model ANOVA with group as the between-participant factor (scenes and letters) and task context as the within-participant factor (living room, bedroom, friend's and school) showed no main effect for either task context (proportion error: *F*_(3,114)_ = 1.53, *p* = 0.21, η_p_^2^ = 0.04; RT: *F*_(3,114)_ = 1.93, *p* = 0.13, η_p_^2^ = 0.05) or group (proportion error: *F*_(1,38)_ < 0.01, *p* = 0.96, η_p_^2^ = 6.7e-5; RT: *F*_(1,38)_ = 0.01, *p* = 0.91, η_p_^2^ = 3.5e-4). For proportion error, the interaction between group and task context was also not significant (*F*_(3,114)_ = 2.20, *p* = 0.09, η_p_^2^ = 0.06). For RTs, there was a significant interaction, (*F*_(3,114)_ = 10.78, *p* = 3.1e-6, η_p_^2^ = 0.22), reflecting relatively fast responses for the school context in the scenes group, but the opposite in the letters group.

### fMRI: univariate

#### ROI analysis

**Figure 4.**
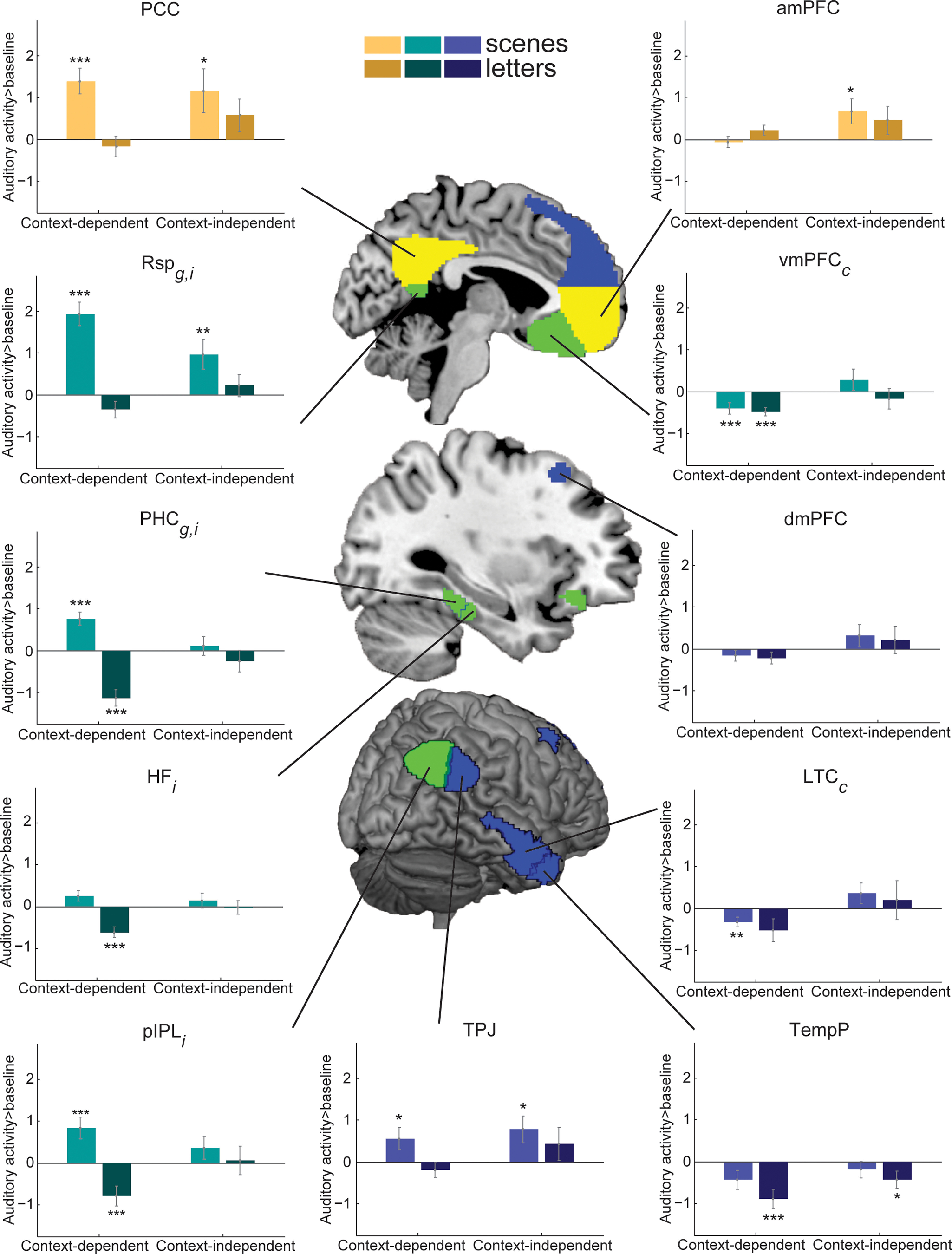
DMN ROI univariate activity estimates during auditory decisions. Beta estimates for auditory responses versus implicit baseline in DMN ROIs, separated by context dependence (context-dependent or context-independent) and group (scene or letter). Asterisks on bars indicate significant differences from baseline (single-sample two-tailed *t* tests) after Holm-Bonferroni multiple comparison correction. ***p < 0.01. **p < 0.02. *p < 0.05. Error bars indicate SEM across participants. For each ROI, subscript letters indicate significant effects from the group × context dependence ANOVA (Table 2), after Holm-Bonferroni multiple correction comparison across ROIs within each network: g, Main effect of group; c, main effect of context dependence; i, interaction. Color represents subnetwork. Yellow represents Core DMN. Green represents MTL DMN. Blue represents dmPFC DMN. Abbreviations as in Table 2.

Separately for each DMN ROI, and for scene and letters groups, Figure 4 plots responses to context-dependent sounds (averaged across types) and the context-independent sound (birdsong). Across DMN regions, two distinct patterns of univariate activity can be seen. In the PCC, Rsp, PHC, HF, and pIPL, a clear preference for scenes is apparent, with this group difference particularly evident for context-dependent sounds (Fig. 4, left column). Interestingly, within the scenes group, these regions show greater activity for the harder context-dependent compared with the easier context-independent decisions. In the dmPFC, amPFC, vmPFC, and LTC, there is little evidence of a group effect; instead, an effect of context dependence is seen, with greater activity in the easier, context-independent condition compared with the context-dependent condition (Fig. 4, right column). The TPJ and TempP appear intermediate with elements of both behaviors.

Data were examined using a three-way mixed-model ANOVA with the factors of ROI, group, and context dependence. Significant main effects of ROI (*F*_(10,38)_ = 10.20, *p* = 3.0e-15, η_p_^2^ = 0.21), group (*F*_(1,38)_ = 6.20, *p* = 0.02, η_p_^2^ = 0.14), and context dependence (*F*_(1,38)_ = 6.02, *p* = 0.02, η_p_^2^ = 0.14) were found, as well as significant interactions between ROI and group (*F*_(10,38)_ = 4.48, *p* = 5.0e-6, η_p_^2^ = 0.11), ROI and context dependence (*F*_(10,38)_ = 5.09, *p* = 5.6e-7, η_p_^2^ = 0.12), context dependence and group (*F*_(1,38)_ = 4.09, *p* = 0.05, η_p_^2^ = 0.10), and a significant three-way interaction between ROI, group, and context (*F*_(10,38)_ = 11.97, *p* = 5.1e-18, η_p_^2^ = 0.24). For each individual ROI, two-way mixed-model ANOVAs with the factors group and context dependence were then performed, with Holm-Bonferroni correction for multiple comparisons across ROIs. The results, presented in Table 2, confirm the above impressions. Medial frontal and anterior/lateral temporal regions only showed a main effect of context dependence, significant after multiple comparison correction in vmPFC and LTC. In comparison, several posterior DMN regions (HF, PHC, Rsp, pIPL) showed a significant interaction between group and context dependence, with PHC and Rsp also showing a main effect of group. Also shown in Figure 4 are the results of *t* tests for each contrast against baseline, similarly corrected for multiple comparisons across ROIs.

**Figure 5.**
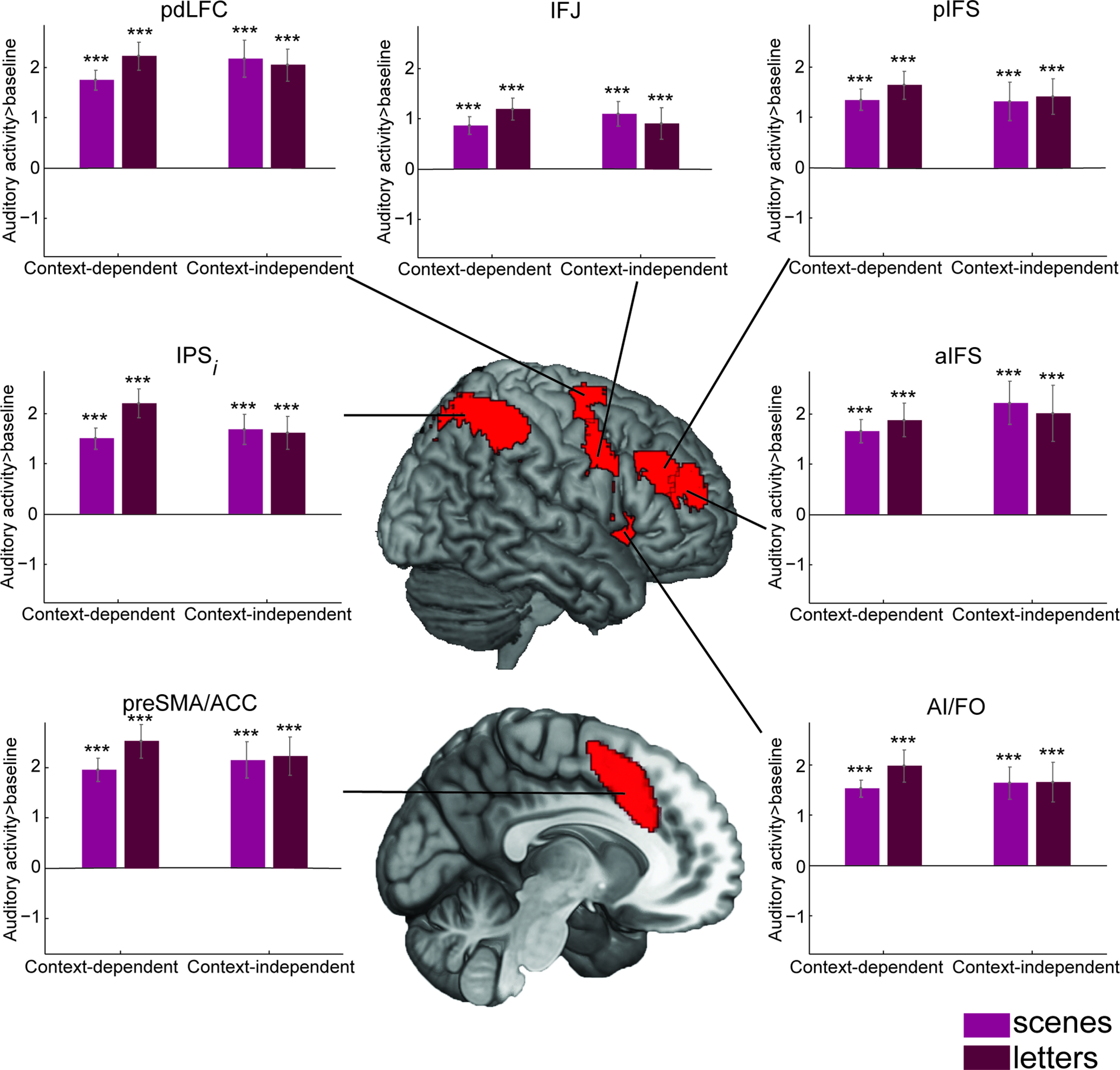
MD ROI univariate activity estimates during auditory decisions. Beta estimates for auditory responses versus implicit baseline in MD ROIs, separated by context dependence (context-dependent or context-independent) and group (scene or letter). Format as in Figure 4. Abbreviations as in Table 2.

The same analyses were repeated for regions of the MD network. For each MD ROI, and for scene and letters groups, Figure 5 plots the BOLD response to context-dependent sounds and the context-independent sound. In contrast to DMN regions, all MD regions showed strong responses for all conditions of the auditory task compared with baseline email activity. For the letters group, as expected, response appeared stronger for the harder context-dependent sounds. Intriguingly, this result was eliminated or even reversed in the scenes group. A three-way mixed-model ANOVA between ROI, group, and context dependence showed a main effect of ROI (*F*_(6,38)_ = 19.45, *p* = 2.7e-18, η_p_^2^ = 0.34) and interaction between ROI and context dependence (*F*_(6,38)_ = 6.12, *p* = 6.2e-6, η_p_^2^ = 0.14). The interaction between group and context dependence also approached significance (*F*_(1,38)_ = 3.94, *p* = 0.05, η_p_^2^ = 0.09). In separate two-way mixed-model ANOVAs for each ROI (Table 2), after correcting for multiple comparisons, the interaction between group and context dependence was significant only in the IPS.

To directly test for a difference between networks, we ran a three-way mixed-model ANOVA with within-participant factors of network (DMN, MD) and context dependence (context-dependent, context-independent), and a between-participant factor of group (scenes, letters). A significant main effect of network (*F*_(1,38)_ = 101.9, *p* = 2.6e-12, η_p_^2^ = 0.73) was found, along with significant interactions between network × context dependence (*F*_(1,38)_ = 10.33, *p* = 2.7e-3, η_p_^2^ = 0.21), and critically, network × group (*F*_(1,38)_ = 6.14, *p* = 0.02, η_p_^2^ = 0.14) and network × group × context dependence (*F*_(1,38)_ = 25.21, *p* = 1.2e-5, η_p_^2^ = 0.40). Neither the main effect of context dependence (*F*_(1,38)_ = 1.84, *p* = 0.18, η_p_^2^ = 0.05) or group (*F*_(1,38)_ = 0.54, *p* = 0.47, η_p_^2^ = 0.01), nor the context dependence × group interaction (*F*_(1,38)_ = 7.2e-3, *p* = 0.93, η_p_^2^ = 1.9e-4) was significant.

#### Whole brain

**Figure 6.**
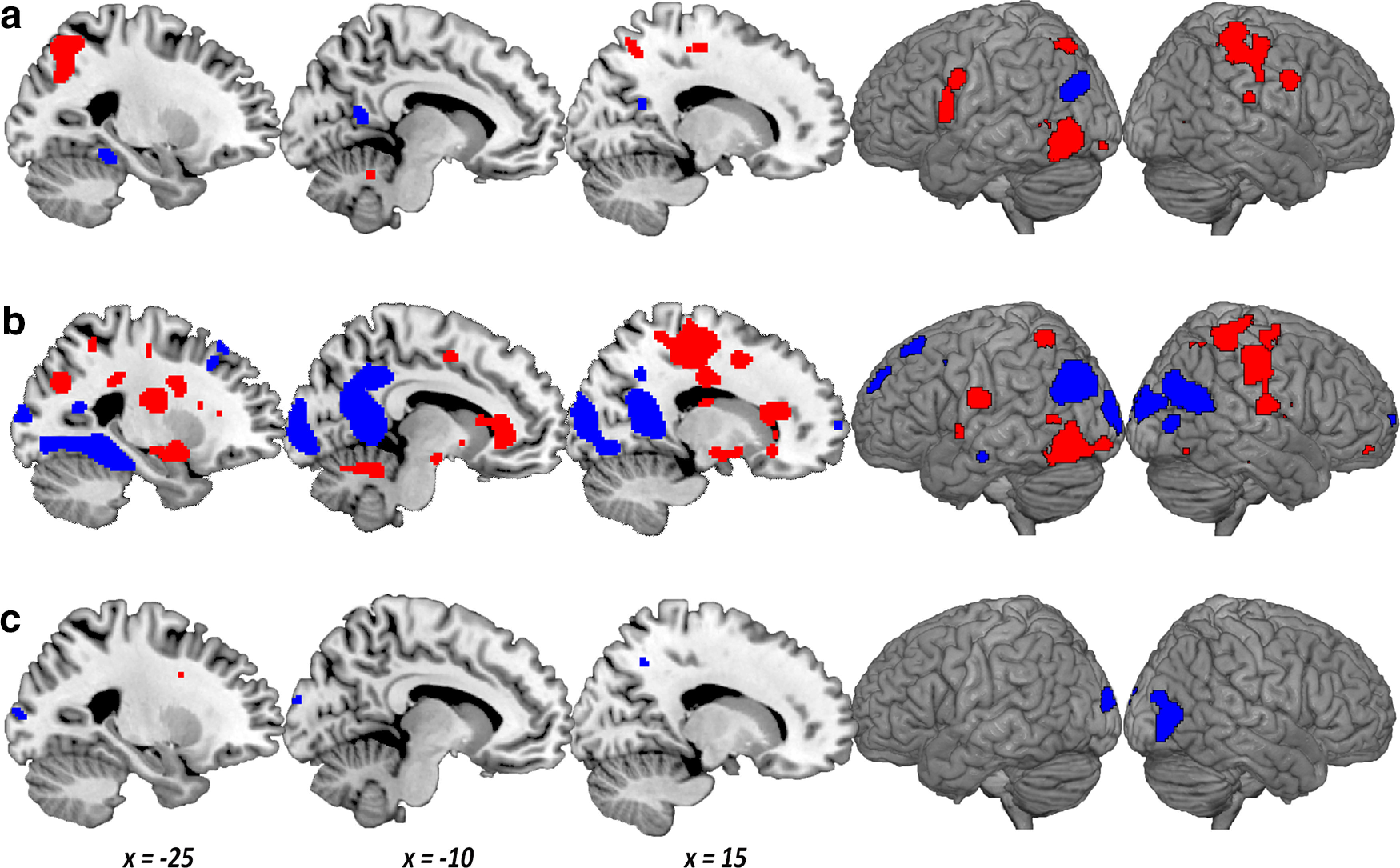
Whole-brain results for (***a***) the interaction of group (scenes, letters) by context dependence (context-dependent, context-independent) (*p* < 0.05, FDR-corrected), (***b***) the simple effect of group for context-dependent sounds only (*p* < 0.01, uncorrected), and (***c***) the simple effect of group for context-independent sounds only (*p* < 0.01, uncorrected). ***a***, Blue represents a more positive scene > letter difference for the context-dependent sounds. Red represents the reverse. ***b***, ***c***, Blue represents voxels in which scenes > letters. Red represents voxels in which letters > scenes. The brain render shows a search depth of 12 voxels. Sagittal slices show *x* coordinate values in MNI space.

To supplement the ROI analyses, Figure 6*a* shows the results of a whole-brain analysis for the interaction between group and context dependence, thresholded at *p* < 0.05, FDR-corrected. To further understand what is driving the interaction, Figure 6*b* shows the simple effect of group for context-dependent sounds and Figure 6*c* shows the simple effect of group for context-independent sounds, thresholded at *p* < 0.01 uncorrected. In line with the ROI analysis (Fig. 4), posterior DMN regions of Rsp, left PHC, and left pIPL showed scenes > letters for context-dependent sounds (Fig. 6*a*,*b*, blue). In contrast, context-dependent sounds showed letters > scenes in IPS and IFJ, along with left extrastriate and right motor cortex (Fig. 6*a*,*b*, red). There was little group difference for context-independent sounds (Fig. 6*c*).

### fMRI: multivariate

**Figure 7.**
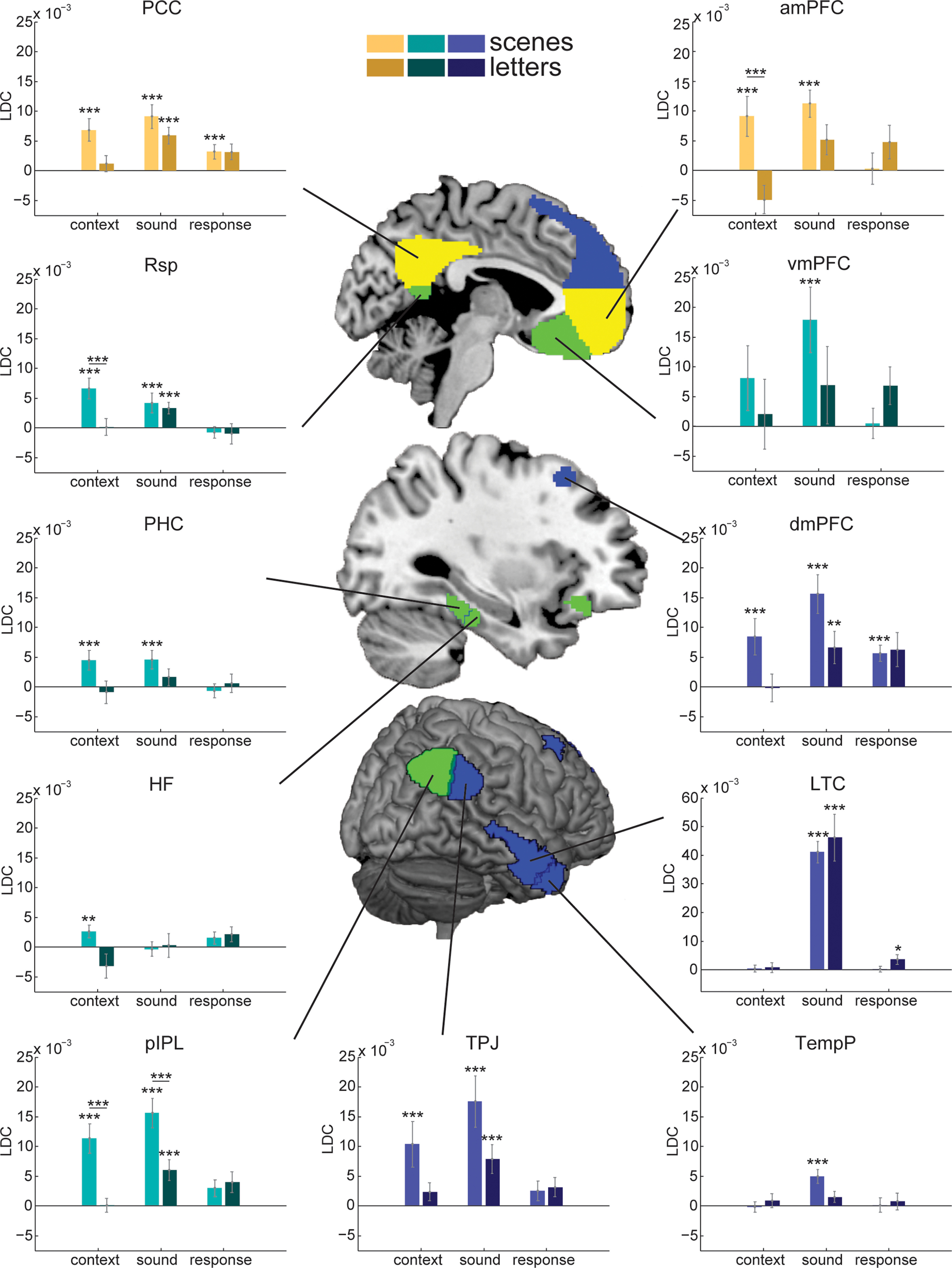
Representational dissimilarity analysis for DMN ROIs during auditory decisions. Asterisks on bars indicate significant above chance decoding (single-sample one-tailed *t* tests). Lines indicate significant group differences (independent-samples two-tailed *t* tests). All *t* tests Holm-Bonferroni-corrected for multiple comparisons across ROIs. ***p < 0.01. **p < 0.02. *p < 0.05. Error bars indicate SEM across participants. Color indicates subnetwork: yellow represents Core DMN; green represents MTL DMN; blue represents dmPFC DMN. Abbreviations as in Table 2.

To understand the representational content in DMN and MD regions, an RSA was performed. Separate contrasts were created to quantify multivariate discrimination of context, sound, and response (see Fig. 2). The analysis was conducted for just the context-dependent sounds, for which context, sounds, and responses were fully crossed. Results for each DMN ROI are shown in Figure 7.

For context decoding, most ROIs showed a difference between groups, with scene contexts decoded more strongly than letter contexts. Data were examined using a two-way mixed-model ANOVA, with group as the between-participant factor and ROI as the within-participant factor. There was a significant main effect of group (*F*_(1,38)_ = 10.48, *p* = 2.5e-3, η_p_^2^ = 0.22); however, the main effect of ROI (*F*_(10,38)_ = 1.91, *p* = 0.42, η_p_^2^ = 0.05) and the interaction (*F*_(10,38)_ = 1.78, *p* = 0.06, η_p_^2^ = 0.05) were not significant. Figure 7 shows results of individual *t* tests, comparing decoding level against chance (0) and independent-samples *t* tests comparing between groups. Again, these *t* tests were Holm-Bonferroni-corrected for multiple comparisons across DMN ROIs. They suggest significant context decoding for scenes in 8 of 11 regions of the DMN. Furthermore, in the Rsp, amPFC, and pIPL, independent-samples *t* tests showed significantly stronger context decoding in the scene compared with the letters group.

Most DMN regions also showed significant sound decoding, which was extremely strong in the lateral temporal ROI (Fig. 7, note scale), presumably because of its proximity to auditory cortex. Like context decoding, sound decoding also tended to be stronger in the scenes group than the letters group with all but the HF showing significant sound decoding in the scenes group. A two-way mixed-model ANOVA showed a significant main effect of ROI (*F*_(10,38)_ = 29.10, *p* = 2.5e-51, η_p_^2^ = 0.43), with neither the effect of group (*F*_(1,38)_ = 2.24, *p* = 0.14, η_p_^2^ = 0.06) nor the interaction significant (*F*_(10,38)_ = 1.43, *p* = 0.16, η_p_^2^ = 0.04). As this analysis could be distorted by the disproportionately strong results of the LTC, this two-way ANOVA was repeated with the LTC ROI removed. Again, there was a main effect of ROI (*F*_(9,38)_ = 5.08, *p* = 2.1e-6, η_p_^2^ = 0.12); and now, the effect of group was also significant (*F*_(1,38)_ = 4.15, *p* = 0.05, η_p_^2^ = 0.10), reflecting greater sound decoding for the scenes group. Independent-samples *t* tests further showed that sound decoding was stronger in the scenes group compared with the letters group in the pIPL, after correcting for multiple comparisons.

A similar ANOVA for response decoding showed only a significant effect of ROI (*F*_(10,38)_ = 2.87, *p* = 1.9e-3, η_p_^2^ = 0.07); the group effect (*F*_(1,38)_ = 1.52, *p* = 0.23, η_p_^2^ = 0.04) and the interaction (*F*_(10,38)_ = 0.86, *p* = 0.57, η_p_^2^ = 0.02) were both nonsignificant. After correcting for multiple comparisons, *t* tests showed significant response decoding in the PCC, dmPFC, and LTC only, with no difference between groups in any ROI.

**Figure 8.**
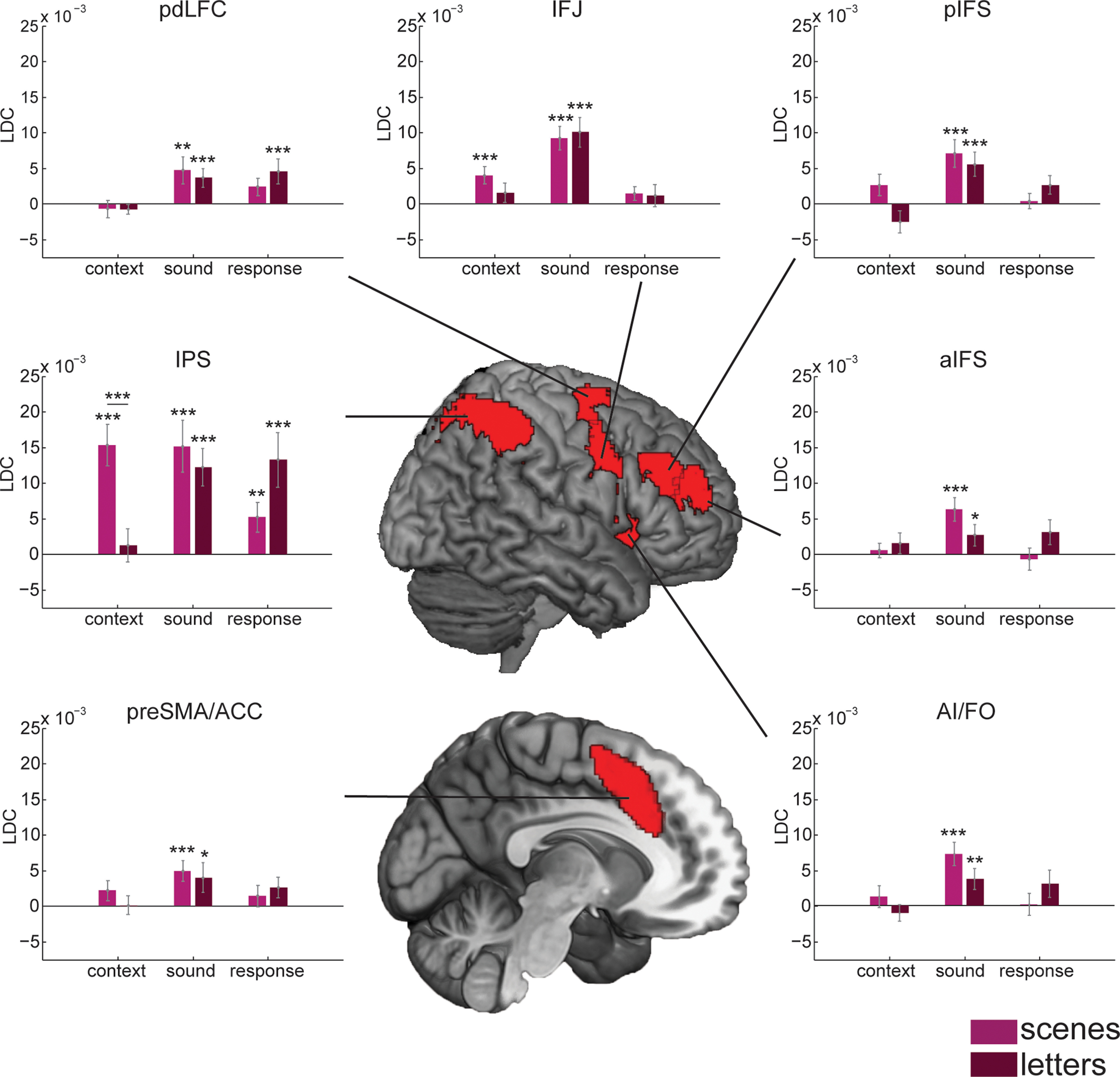
Representational dissimilarity analysis for MD ROIs during the auditory decisions. Format as in Figure 7. Abbreviations as in Table 2.

Figure 8 presents the results of similar analyses for MD ROIs. Again, a two-way mixed-model ANOVA was constructed for each type of information, with group as the between-participant factor and ROI as the within-participant factor. For context decoding, a few MD regions, especially the IPS, showed stronger decoding of scenes than of letters. ANOVA showed significant main effects of ROI (*F*_(6,38)_ = 10.25, *p* = 4.8e-10, η_p_^2^ = 0.21) and group (*F*_(1,38)_ = 7.57, *p* = 9.0e-3, η_p_^2^ = 0.17), along with a significant interaction (*F*_(6,38)_ = 6.85, *p* = 1.1e-6, η_p_^2^ = 0.15). Additional *t* tests, Holm-Bonferroni-corrected for multiple comparisons across MD ROIs, revealed significant context decoding for scenes in IPS and IFJ, and significantly stronger scene than letter decoding in IPS. All regions showed strong sound decoding with minimal difference between groups. Again, decoding was particularly strong in IPS. The two-way mixed-model ANOVA showed a significant main effect of ROI (*F*_(6,38)_ = 10.68, *p* = 1.9e-10, η_p_^2^ = 0.22), while the effect of group (*F*_(1,38)_ = 0.85, *p* = 0.36, η_p_^2^ = 0.02) and the interaction were not significant (*F*_(6,38)_ = 0.54, *p* = 0.77, η_p_^2^ = 0.01). Even after correcting for multiple comparisons, *t* tests showed significant sound decoding for both sound and letters groups across all MD regions, with no effects of group in any region. Response decoding was particularly strong in IPS. Again, the ANOVA showed a significant effect of ROI (*F*_(6,38)_ = 7.67, *p* = 1.6e-7, η_p_^2^ = 0.17), while the group effect (*F*_(1,38)_ = 2.92, *p* = 0.10, η_p_^2^ = 0.07) and the interaction (*F*_(6,38)_ = 1.55, *p* = 0.16, η_p_^2^ = 0.04) were not significant. *t* tests showed response decoding only in IPS and posterior dorsal region of lateral PFC, with no difference between groups in any ROI.

### Effect of within-participant RT differences

To examine the potential contribution of RT differences to the RSA results, the same contrast analysis was repeated with RT data. The results showed no differences between the scenes and letters groups for context (*t_(_*_38)_ = 0.11, *p* = 0.91, *d* = 0.04), sound (*t*_(38)_ = 0.62, *p* = 0. 54, *d* = 0.20), or response (*t*_(38)_ = 0.67, *p* = 0. 50, *d* = 0.21) contrasts. Furthermore, RT differences were not significantly greater than zero for context decoding (scenes: *t*_(19)_ = 1.35, *p* = 0.19, *d* = 0.30, letters: *t*_(19)_ = 1.31, *p* = 0.21, *d* = 0.29) or response decoding in the scenes group (*t*_(19)_ = 1.13, *p* = 0.27, *d* = 0.25). RT differences greater than zero were observed for sound decoding (scenes: *t*_(19)_ = 3.58, *p* = 2.0e-3, *d* = 0.80, letters: *t*_(19)_ = 8.63, *p* = 5.3e-8, *d* = 1.93) and for response decoding in the letters group (*t*_(19)_ = 2.23, *p* = 0.04, *d* = 0.50). Thus, group differences in the fMRI RSA were not mirrored by corresponding differences in the RT analysis.

### Context decoding at block onset

To determine whether context information is processed at the time of context change as well as during the auditory task, context decoding was examined at block onset. As explained in Materials and Methods, on 3/4 of blocks, the context type changed compared with the previous block. On the other 1/4 of blocks, the context stayed the same as the previous block. These block onsets were modeled separately, creating context switch and context stay block onset regressors. If context information is processed at the time of a context switch, then we would expect to see context decoding after a context switch more so than after a context stay. To test this, for each network, a three-way mixed-model ANOVA was created, with group as the between-participant factor and ROI and context switch type (context switch, context stay) as within-participant factors.

For the DMN, unlike response to the auditory stimulus, ANOVA showed no significant main effects (group: *F*_(1,38)_ = 0.06, *p* = 0.81, η_p_^2^ = 1.6e-3; ROI: *F*_(10,380)_ = 1.00, *p* = 0.44, η_p_^2^ = 0.03; context switch type: *F*_(1,38)_ = 7.6e-3, *p* = 0.93, η_p_^2^ = 2.0e-4) or interactions (ROI × switch type: *F*_(10,380)_ = 1.76, *p* = 0.07, η_p_^2^ = 0.04; ROI × group: *F*_(10,380)_ = 0.51, *p* = 0.89, η_p_^2^ = 0.01; switch type × group: *F*_(1,38)_ = 1.11, *p* = 0.30, η_p_^2^ = 0.03; ROI × switch type × group: *F*_(10,380)_ = 0.18, *p* = 0.99, η_p_^2^ = 4.7e-3). Averaging across DMN ROIs, context switch type and group, a one-sample *t* test found that overall context decoding was not significantly greater than chance (*t*_(39)_ = 0.65, *p* = 0.52, *d* = 0.10). Furthermore, individual *t* tests for each ROI, separately for each context switch type and group, showed no significant context decoding after Holm-Bonferroni correction for multiple comparisons across DMN ROIs.

Results were similar for the MD network, with no significant main effects (group: *F*_(1,38)_ = 0.03, *p* = 0.88, η_p_^2^ = 6.6e-4; ROI: *F*_(6,228)_ = 0.34, *p* = 0.92, η_p_^2^ = 8.9e-3; context switch type: *F*_(1,38)_ = 0.06, *p* = 0.81, η_p_^2^ = 1.5e-3), or interactions (ROI × switch type: *F*_(6,228)_ = 1.27, *p* = 0.27, η_p_^2^ = 0.03; ROI × group: *F*_(6,228)_ = 0.97, *p* = 0.45, η_p_^2^ = 0.03; switch type × group: *F*_(1,38)_ = 0.76, *p* = 0.39, η_p_^2^ = 0.02; ROI × switch type × group: *F*_(6,228)_ = 0.75, *p* = 0.61, η_p_^2^ = 0.02). Averaging across MD ROIs, context switch type and group, a one-sample *t* test found that context decoding was not significantly greater than chance (*t*_(1,39)_ = 0.90, *p* = 0.38, *d* = 0.14). Individual *t* tests for each ROI, separately for each context switch type and group, with Holm-Bonferroni correction for multiple comparisons across MD ROIs, showed only 1 case of significant context decoding (IPS, context switch, scenes group, *t*_(19)_ = 3.66, *p* = 8.3e-4, *d* = 0.82).

## Discussion

This experiment tested the hypothesis that the DMN might be associated with contextual decision-making in life-like contexts. To this end, brain activity during decision-making using life-like scenes was compared with activity during decision-making using symbolic letter cues. DMN regions were hypothesized to respond preferentially for life-like contexts, whereas the MD system was hypothesized to be active for all decisions, regardless of the context type.

Despite minimal behavioral differences between groups, substantial effects of group were found in neural responses of the two networks of interest. In DMN regions, two distinct patterns of univariate responses were identified. Medial prefrontal and anterior/lateral temporal DMN regions showed greater activity for context-independent decisions compared with context-dependent decisions, consistent with the standard DMN finding of reduced activation with increased task difficulty (McKiernan et al., 2003, 2006; Fox et al., 2005; Kelly et al., 2008). This result held for both scene and letter groups. A group of posterior DMN regions, however, were sensitive to context type. In line with theories emphasizing the importance of DMN regions in constructing and representing scenes, events, and situation models (Bar, 2007, 2009; Hassabis and Maguire, 2007; Ranganath and Ritchey, 2012), these posterior DMN regions showed greater activation for scenes compared with letters. Importantly, in the scenes group, activity in these posterior regions was stronger for context-dependent decisions compared with context-independent decisions, reversing the context dependence effects found in the letters group. These findings suggest that posterior DMN regions are important for contextual decision-making in meaningful, life-like contexts.

Interestingly, activation patterns across the DMN network do not fully follow the proposed segmentation into core, MTL, and dmPFC subsystems (Andrews-Hanna et al., 2010; Wen et al., 2020; see also Yeo et al., 2011). Instead, our univariate results suggest a broad dissociation between posterior and anterior DMN regions. Thus, our results are somewhat more compatible with the proposal that posterior HF, PHC, Rsp, posterior cingulate, and pIPL make up a posterior medial system, playing a primary role in the construction and representation of a situation model (Ranganath and Ritchey, 2012). These situation models are thought to hold information about the broad features of the current situation and relevant information associated with these situational features. In our data, these were the regions showing especially strong activity when background scenes controlled context-dependent decisions. One exception is that, although the vmPFC is also described as part of the posterior medial system (Ranganath and Ritchey, 2012), in these data, its profile matched anterior/lateral temporal regions in showing no preference for scenes. Thus, while medial frontal cortex may couple with medial parietal cortex at rest (Andrews-Hanna et al., 2010), it appears to have different functional preferences, consistent with other observations (e.g., Wen et al., 2020).

In multivariate analysis, there was less distinction between DMN ROIs, with stronger encoding of scenes than of letter contexts in most DMN regions. Across the whole DMN, furthermore, there was a general trend for stronger sound encoding in scene than in letter contexts. Therefore, although regions of the posterior medial system may be especially central to scene-based decision-making, our data also give evidence for some involvement of the whole DMN.

As posterior DMN regions have been repeatedly linked to visual scene perception (Epstein and Kanwisher, 1998; Bird and Burgess, 2008; Lee et al., 2008), one might wonder whether DMN activity during context-dependent decision-making in the scenes group is simply related to visual scene perception, necessary only when processing of the scene is required to make a decision, rather than also representing contextual associations required to make context-dependent decisions. Although a definitive answer would require additional experiments with nonvisual contexts, several aspects of the findings suggest that this may not be the entire story. First, context decoding during the auditory task, especially in the scenes group, extends to anterior regions of the DMN as well as posterior regions more usually related to scene processing. Second, context decoding in DMN regions was not found with a scene change, where a large visual change is likely to drive some automatic processing of the scene, and was instead only found when participants were required to use the context to make context-dependent decisions. More proactive context representation may be seen in other situations, for example, if participants had not been focused on the ongoing email task. The lack of a group difference in context coding at the time of changes could also, along with other null results, reflect limited power given the sample size. Furthermore, many DMN regions were also found to represent sound type as well as context and, across these regions, sound representation was also enhanced in the scenes group. These results suggest that, in scene-based decision-making, DMN regions represent not just the scenes themselves, but also the contents of the decision that the scene context controls.

Scenes and letter cues had many visual differences, with the scenes containing complex images extending across a large area. It seems likely, however, that visual differences alone do not explain the involvement of the DMN in scene-based decision-making. Instead, our findings likely reflect the well-established role of the DMN in retrieving and using episodic and semantic knowledge (Addis et al., 2007; Andrews-Hanna et al., 2010; Vilberg and Rugg, 2012).

Research from the cognitive control literature would suggest that MD regions are important for task control across many cognitive demands (Duncan and Owen, 2000; Duncan, 2010, 2013; Fedorenko et al., 2013). Correspondingly, MD regions showed a strong increase in activity during auditory decision-making events compared with the baseline email task in both scene and letter groups. However, contrary to the typical increase in MD activity with increased task demands (Fedorenko et al., 2013), MD regions did not show a simple increase in activity for context-dependent compared with context-independent decisions. Instead, for the scene group only, the usual result was reversed, with stronger activation for a context-independent decision. Importantly, this interaction was the reverse of that shown in posterior DMN regions, where activation increased for context-dependent sounds compared with context-independent birdsong in the scenes group. The results suggest that, during decision-making based on a rich meaningful context, it is DMN, rather than MD, regions that reflect context-dependent decision-making processes.

In MD regions, context representation was generally weak, restricted to significant scene decoding in IPS and IFJ. At first glance, the context decoding results for MD regions, showing no letter context decoding, seem contrary to the univariate results, showing the relative importance of MD regions for letter-based decisions. However, in the letters group, the context-dependent task did not require representation of the entire letter pair on any given trial. Instead, when a sound was played, the participant could simply check whether its first letter was included in the letter pair at the top of the screen. As the letter searched for was related to the played sound, letter processing may largely have been reflected in our measure of sound decoding. Unlike the DMN, the MD network showed similar sound decoding in scene and letter groups.

Recently, Margulies et al. (2016) proposed that the cortex can be organized along a principle gradient from unimodal regions, serving primary sensory or motor functions, to regions of transmodal cortex, thought to represent memory-based heteromodal concepts. This principle gradient captured the spatial organization of the seven functional connectivity networks identified by Yeo et al. (2011) with DMN at the top of this gradient, and executive-control networks, such as MD, in between the DMN and sensory control networks. This topographical organization of the cortex, Margulies et al. (2016) suggested, indicates a role for the DMN in tasks requiring a rich representation of stimuli, linked to internal, memory-based representations, beyond their immediate sensory properties. In support of this, Murphy et al. (2018) found that variance in brain activity during a decision-making task driven by conceptual information held in memory was captured by this principle gradient, with increased activity from sensorimotor regions to transmodal regions. The results of our experiment could also be considered in terms of this processing hierarchy, with the DMN important for decisions requiring semantic associations afforded by the current scene.

Linking together research on situation models, episodic memory recall and contextual cognitive control, these findings suggest that DMN regions can play a role in context-dependent decision-making, but primarily when the context is based on rich semantic associations rather than abstract symbols. Under these circumstances, posterior regions of the DMN show univariate activity at the time of the decision, whereas a more extensive DMN network shows multivariate coding of both the scene context itself and the contents of the decision it controls. Results for MD regions are different, with strong univariate activity whenever a decision is required, and, intriguingly, somewhat reduced activity in the case of demanding decisions that depend on scene context. Depending on context, we suggest that either DMN or MD regions may play a prominent role in selection and control of appropriate behavior.
